# Emotional Reactions to Cybersecurity Breach Situations: Scenario-Based Survey Study

**DOI:** 10.2196/24879

**Published:** 2021-05-12

**Authors:** Sanja Budimir, Johnny R J Fontaine, Nicole M A Huijts, Antal Haans, George Loukas, Etienne B Roesch

**Affiliations:** 1 Department of Work, Organization and Society Faculty of Psychology and Educational Sciences Ghent University Ghent Belgium; 2 Department for Psychotherapy and Biopsychosocial Health Faculty of Health and Medicine Danube University Krems Krems an der Donau Austria; 3 Eindhoven University of Technology Eindhoven Netherlands; 4 University of Greenwich London United Kingdom; 5 Centre for Integrative Neuroscience and Neurodynamics School of Psychology and Clinical Language Sciences University of Reading Reading United Kingdom

**Keywords:** cybersecurity breach victims, emotions, personality, mental health, Internet of Things

## Abstract

**Background:**

With the ever-expanding interconnectedness of the internet and especially with the recent development of the Internet of Things, people are increasingly at risk for cybersecurity breaches that can have far-reaching consequences for their personal and professional lives, with psychological and mental health ramifications.

**Objective:**

We aimed to identify the dimensional structure of emotion processes triggered by one of the most emblematic scenarios of cybersecurity breach, the hacking of one’s smart security camera, and explore which personality characteristics systematically relate to these emotion dimensions.

**Methods:**

A total of 902 participants from the United Kingdom and the Netherlands reported their emotion processes triggered by a cybersecurity breach scenario. Moreover, they reported on their Big Five personality traits, as well as on key indicators for resilient, overcontrolling (internalizing problems), and undercontrolling (aggression) personality types.

**Results:**

Principal component analyses revealed a clear 3-dimensional structure of emotion processes: emotional intensity, proactive versus fight/flight reactions, and affective versus cognitive/motivational reactions. Regression analyses revealed that more internalizing problems (β=.33, *P*<.001), resilience (β=.22, *P*<.001), and agreeableness (β=.12, *P*<.001) and less emotional stability (β=–.25, *P*<.001) have significant predictive value for higher emotional intensity. More internalizing problems (β=.26, *P*<.001), aggression (β=.25, *P*<.001), and extraversion (β=.07, *P*=.01) and less resilience (β=–.19, *P*<.001), agreeableness (β=–.34, *P*<.001), consciousness (β=–.19, *P*<.001), and openness (β=–.22, *P*<.001) have significant predictive value for comparatively more fight/flight than proactive reactions. Less internalizing problems (β=–.32, *P*<.001) and more emotional stability (β=.14, *P*<.001) and aggression (β=.13, *P*<.001) have significant predictive value for a comparatively higher salience for cognitive/motivational than affective reactions.

**Conclusions:**

To adequately describe the emotion processes triggered by a cybersecurity breach, two more dimensions are needed over and above the general negative affectivity dimension. This multidimensional structure is further supported by the differential relationships of the emotion dimensions with personality characteristics. The discovered emotion structure could be used for consistent predictions about who is at risk to develop long-term mental well-being issues due to a cybersecurity breach experience.

## Introduction

### Background

The increasing number of Internet of Things devices (IoT) and their diverse application in private and work lives offer unlimited possibilities for a connected life. However, this has also extended the scope of security breaches and cybercriminal behavior [1]. As cyberattacks became more and more focused on specific companies and individual users [2,3], they increasingly create technological, economic, social, and psychological challenges. Because of the deep penetration of IoT in personal lives, cybersecurity breaches on such devices can have far-reaching personal consequences. Work and livelihood can be disturbed, personal and social spheres can be altered, and these changes can sometimes be irrevocable. The most direct psychological effects of such events, which are intrinsically relevant to the personal goals of the user, are the emotional responses they elicit [4,5]. A leading security company reported that negative emotions including anger, annoyance, frustration, upset, and a feeling of being cheated are the common reactions to being a victim of cybercrime [6]. These emotional experiences could develop into long-term, far-reaching psychological turmoil [7-10]. Despite their central role in psychological well-being, very little is known about emotional reactions in the context of cybersecurity breaches. In this study, we (1) explore victims’ emotion processes by employing a scenario study with a cybersecurity breach on a smart security camera, which is one of the most telling examples of invasion of privacy by unauthorized entrance in the private sphere [11,12], (2) explore which personality characteristics predict interindividual differences in emotional reactions to this cybersecurity breach; and (3) designed the explorative research in such a way to generate replicable findings.

### Emotion Processes

In emotion research, participants are often asked to report on their own emotions by evaluating emotion and affect terms (eg, the frequently used Positive and Negative Affect Schedule [PANAS] [13]). While this type of research can generate very interesting findings, it does not allow researchers to unearth the emotion processes these affect terms refer to. To get a comprehensive view of the emotion processes that can be elicited by cybersecurity breaches, emotions are currently studied on the basis of the componential emotion approach [14]. This approach has emerged as an overarching conceptual framework within the scientific field of emotion research. According to this approach, emotions are conceptualized as processes that are elicited by goal-relevant events and consist of an interplay between 5 major components: appraisals, action tendencies, bodily responses, expressions, and subjective feelings [5]. Each component has a function. Appraisals are the evaluation of the eliciting event against one’s goals, needs, and values. Action tendencies refer to the preparation and direction of adaptive action. Bodily responses refer to physiological changes that prepare the body for actual action. Expressions are the facial, vocal, and gestural reactions through which the ongoing emotion process is communicated. Through subjective feelings, the individual becomes aware of the ongoing emotion process. These feelings are often communicated with the use of emotion and affect terms. Moreover, emotion processes are evolutionary-shaped processes that have evolved from reflex-like reactions to dynamic processes open to regulation [5,14]. All aspects of the emotion process can be regulated, from the impulsive reactions to the cognitive evaluations. Having flexible emotion processes allows us to better adapt to our environment [15].

This componential emotion approach is especially promising for studying emotional experiences, as it has been demonstrated across cultural and linguistic groups that the 5 components, as well as regulation are encoded in daily language. First in 3 samples from the United Kingdom, Switzerland, and Belgium [16] and later in 31 additional samples from 24 countries and representing 20 languages (such as Chinese and Japanese) [14], it was demonstrated that 142 emotion features representing the 5 emotion components and regulation systematically constitute the meaning of 24 frequently used emotion terms [14]. The componential emotion approach forms not only a comprehensive theoretical framework but also represents how people naturally think and talk about their emotions.

Thus, to fully understand emotion dynamics, it is important to go beyond feeling and emotion terms and study all emotion components and regulation processes. In this study, the dimensions that structure the emotion processes elicited by a cybersecurity breach of a smart security camera are exploratively identified by taking all emotion components as well as regulation into account.

### Person Characteristics and Emotional Reactions

To better understand the emotion dimensions involved in this scenario, we evaluate whether and how characteristics of personality are related to the reported emotional experience. To this end, we have worked with 2 broad personality models that have been shown in the past to relate to emotional functioning: the Big Five personality model [17] and the resilient/overcontrolled/undercontrolled personality type model [18,19].

#### Big Five Personality Model

In the first model, personality is described by the Big Five broad personality traits: extraversion, emotional stability, conscientiousness, agreeableness, and openness [17]. These traits have been shown to relate to the duration of emotional states and frequency of specific emotional experiences [20]. A very common finding is that extraversion is positively associated with positive affect and emotional stability negatively with negative affect [21]. Additionally, associations of personality traits with emotion regulation were demonstrated in several studies [22-24]. For instance, extraversion, conscientiousness, and openness were predictive for problem solving and cognitive restructuring, while agreeableness was predictive for social support and cognitive restructuring [22-24].

#### Resilient/Overcontrolled/Undercontrolled Personality Type Model

The second personality model classifies people into 3 broad personality types [18,19]. Resilient people are characterized by a tendency to effectively adapt to changes and have the ability to recover well from stress and negative emotional arousal. Overcontrolled people are introverted and emotionally sensitive but also dependable. They are more likely to experience sadness and fear and are at risk of developing internalizing complaints such as depression and anxiety. Undercontrolled people are low on agreeableness and conscientiousness and high on aggressiveness and delinquency. They are more likely to experience anger and are at risk to develop externalizing problems.

#### Gender and Age

Next to these personality predictors, we have also looked at the relationships with gender and age. Overall gender differences in emotional reactions have been observed, with females having more intense emotional reactions compared with males [25-28]. Regarding age, a general decrease of negative affective experiences [29] and increase of healthier emotion regulation strategies [30-32] have been observed throughout the life span.

### Replicability

In light of the replicability crisis in psychology [33] and because of the explorative nature of this research with the innovation to root the study of emotional experiences in the componential emotion approach, the study was executed in two samples from different countries (United Kingdom and the Netherlands) speaking different languages (English and Dutch). Moreover, participants in each country received at random one of two versions of the cybersecurity breach scenario. In one version, the smart security camera showed obvious signs of a cybersecurity breach (nonambiguous condition), and in the other version, it showed unclear signs (the ambiguous condition) that could also potentially be caused by other factors (eg, a bug in the software). By adding the latter scenario, the ecological validity of the research was increased, as in daily life it is also often unclear whether or not a dysfunction of internet-connected devices is due to a cybersecurity breach.

## Methods

### Sample

A total of 1045 participants were recruited through Qualtrics panel, 524 participants from the United Kingdom and 521 participants from the Netherlands. Before the data analyses, participants showing signs of not properly answering questions were removed. One of the strongest indicators that the validity of responses is at stake is nondifferentiation of the responses [34]. All participants who gave the same response on at least 75% of the GRID items and on 70% of the International Personality Item Pool 50 (IPIP-50) questionnaire items deviated from most participants in scale use and were removed (n=143 deleted cases). This left 902 participants for the analyses. Sample characteristics are presented in Table 1.

**Table 1:** Study sample characteristics (n=902).

Characteristics			Country of residence, n (%)					Total, n (%)
			United Kingdom (n=435)		Netherlands (n=467)			
**Gender**								
	Female	221 (50.8)		231 (49.5)		452 (50.1)		
	Male	214 (49.2)		236 (50.5)		450 (49.9)		
**Condition**								
	Ambiguous	217 (49.9)		241 (51.6)		458 (50.8)		
	Nonambiguous	218 (50.1)		226 (48.4)		444 (49.2)		

### Procedure

The Qualtrics project team organized and coordinated data collection. They recruited samples from both countries based on their Qualtrics panel of participants. Quotas for samples were predefined and balanced by country of residence, gender, and scenario with age range limited from 18 to 65 years. An online questionnaire, located on the Qualtrics survey platform, was presented to participants remotely by sending them a survey link. Each participant electronically signed an online informed consent form prior to completing the questionnaire. Participants had the opportunity to complete the questionnaire within a 1-week period. The average duration of questionnaire completion was 15 minutes. Each participant was presented with an introduction explaining what IoT devices are and specifically what a smart security camera is. This was followed by the presentation of one of the two scenarios (ambiguous or nonambiguous, see complete instructions in Multimedia Appendix 1). Each participant thus evaluated only randomly assigned one scenario.

### Measures

#### Emotion Assessment

Participants were asked to imagine they experienced one out of two cybersecurity breach scenarios. Scenario 1, which represented the ambiguous condition, was formulated as follows: “Imagine that you bought a smart security camera for your home. After some time, you notice that the shutter on your smart security camera starts opening and closing without your instruction, several times for a few minutes, then it stops for a minute and starts again opening and closing several times and then it stops.” In the nonambiguous condition (scenario 2), the formulation was “Imagine that you bought a smart security camera for your home. After some time, you notice that the shutter on your smart security camera opens without your instruction and the camera rotates toward you and then starts following your movement.”

Participants were subsequently asked to report the emotional reactions they would have in the situation presented using the Cybersecurity GRID questionnaire (Multimedia Appendix 2). This is an adjusted version of the GRID instrument that was used to study the meaning of emotion words across cultural and linguistic groups [14] and is based on the componential emotion approach [5] including the assessment of the 5 emotion components and emotion regulation. In order to determine and operationalize relevant features of the emotion processes in the specific context of cybersecurity breaches, we executed a preliminary qualitative survey. In this survey, 130 participants reported on their real or expected emotional reactions in cybersecurity breach situations (either from first-hand experience or based on a third-party experience). Participants’ reports included a brief description of the cybersecurity breach situation and the emotional reactions they had or would have had in that situation (referring to each of the 5 emotion components and regulation). The new Cybersecurity GRID questionnaire was based on those emotion features that were reported by at least 15% of the participants. The Cybersecurity GRID contains 76 items (19 appraisals, 16 action tendencies, 11 bodily reactions, 8 expressions, 14 subjective feelings, and 8 emotion regulation strategies). Each emotion feature was evaluated on the 7-point Likert scale commonly used in survey research ranging from 1 (strongly disagree) to 7 (strongly agree) [35].

#### IPIP-50

IPIP-50 [17] is a validated instrument that measures the Big Five personality factors. Participants rated how accurately each statement described them on a 5-point Likert scale ranging from 1 (very inaccurate) to 5 (very accurate). Person mean-centered scores were calculated for IPIP items and reversed according to instructions. Each factor showed good to very good internal consistency (Cronbach alpha): extraversion: α=.85, agreeableness: α=.83, conscientiousness: α=.79, emotional stability: α=.83, and for openness: α=.72.

#### Depression, Anxiety, and Stress Scale–21 Item

The Depression, Anxiety, and Stress Scale–21 Item (DASS-21) assesses internalizing problems, which is a key feature of the overcontrolled personality type. It is a shortened 21-item version of the Depression, Anxiety, and Stress Scale [36]. Items are rated on a 4-point scale ranging from 0 (does not apply at all) to 3 (applies very much). The total DASS-21 sum scores showed high internal consistency, α=.96.

#### Short-Form Buss-Perry Aggression Questionnaire

The Short-Form Buss-Perry Aggression Questionnaire [37-39] assesses aggression, which a key feature of the undercontrolled personality type. It is a short version of the Buss-Perry Aggression Questionnaire [40] and consists of 21 items rated on a 5-point scale ranging from 1 (extremely uncharacteristic of me) to 5 (extremely characteristic of me). The scale showed a high internal consistency, α=.92.

#### Ego Resilience Scale

The Ego Resilience Scale [41,42] is a short, revised version of the Ego-Resiliency Scale [43], measuring self-reported resilience on 10 items on a 4-point scale ranging from 1 (does not apply at all) to 4 (applies very strongly). The Cronbach alpha of the total score was .78.

### Ethical Approval

Ethical approval was obtained from the ethical committee of Ghent University, Faculty of Psychology and Educational Sciences in 2017 (number 2016/67).

## Results

### Internal Structure of Emotional Reactions

Principal component analyses were applied to identify the major dimensions of variability among 76 emotion features. To avoid confusion between emotion components from a substantive point of view and principal components obtained from analysis, the latter will be referred to as dimensions in the remainder of the text.

To identify the number of dimensions, 3 criteria were used: (1) the scree plot based on the Eigenvalues (Multimedia Appendix 1), (2) interpretability, and (3) replicability for each language, scenario, and gender (Multimedia Appendix 1). The theoretically best interpretable rotation was selected. A highly stable and well-interpretable 3-dimensional structure was identified that accounted for 48% of the total variance (see Table 2 for the highest loading features on each dimension and Multimedia Appendix 1 for the full loading matrix).

On the first dimension, accounting for 31% of variance, all emotion features have a positive loading, with the subjective experiences loading highest (eg, I would feel panic, I would feel upset). The higher participants score on this dimension, the more intense negative emotion processes are elicited by the scenario. Therefore, this dimension is named emotional intensity.

The second dimension, accounting for 12% of variance, is a bipolar dimension. One pole is defined by proactive action tendencies to deal with the cybersecurity breach (eg, I would want to regain control over the device/account, I would want to find a solution and fix the problem). The other pole is defined by fight/flight action tendencies (eg, I would want to take revenge, I would want to isolate myself physically) and features from other components that indicate distress (eg, I would have pain in the chest). Therefore, this dimension is labeled proactive versus fight/flight.

**Table 2:** Results from principal component analysis of the Cybersecurity GRID questionnaire (n=920)^a^.

GRID items			Dimension loading				
			1		2		3
**Dimension 1: Emotional intensity**							
	SF5. I would feel panic.	.73		.20		–.30	
	SF4. I would feel afraid.	.72		.09		–.26	
	SF7. I would feel worried.	.70		–.15		–.21	
	SF6. I would feel upset.	.70		.04		–.27	
	SF14. I would feel uncomfortable.	.67		–.14		–.12	
	SF11. I would feel angry.	.67		.01		–.15	
**Dimension 2: Proactive versus fight/flight**							
	AT14. I would want to destroy whatever was close.	.32		.65		.10	
	AT15. I would want to take revenge.	.36		.63		.16	
	BR4. I would have pain in the chest.	.49		.61		–.22	
	AT1. I would want to stop what was happening.	.43		–.62		.16	
	AT9. I would want to find a solution and fix the problem.	.34		–.64		.12	
	AT2. I would want to regain control over the device/account.	.44		–.68		.14	
**Dimension 3: Affective versus cognitive/motivational**							
	A19. I would think “It is not safe that this device is connected to the internet.”	.58		–.07		.43	
	A7. I would think “My trust is betrayed.”	.58		.13		.42	
	A12. I would think “It is happening because someone is trying to hack and take control over my count.”	.56		–.10		.41	
	E8. I would be walking around nervously.	.60		.32		–.34	
	E7. I would be restless (touching face, hair, biting nails, nervously kicking with legs).	.58		.34		–.35	
	ER3. I would try to calm myself down (eg, by breathing deeply).	.59		–.09		–.37	

^a^The 6 highest loadings are presented, and the full loading matrix can be found in Multimedia Appendix 1.

The third dimension, accounting for 5% of the variance, is also bipolar. All appraisal and action tendency features (eg, I would think “It is not safe that this device is connected to the Internet”) have a nonnegative loading, while all subjective experience, bodily reaction, expression, and regulation features (eg, I would try to calm myself down) have a nonpositive loading on this dimension. This dimension is labeled affective versus cognitive/motivational.

### Predictors of Emotional Reactions

The scores on each of the 3 identified emotion dimensions were regressed on the personality characteristics. As the Big Five indicators and the resilience, overcontrolled, and undercontrolled indicators show both theoretical and empirical overlap (and the differences and similarities between personality models do not form the focus of this research), their predictive value was investigated separately. Hierarchical linear regression analyses were performed. In the baseline model (model 1), the predictors are country of residence, scenario, gender, and age (with United Kingdom, ambiguous situation, and women being the reference categories). In the second model the personality characteristics were added as predictors: the Big Five personality traits in model 2a and the indicators for resilience, overcontrolled, and undercontrolled personality types in model 2b.

#### Emotional Intensity

In model 1 (Table 3), it was observed that United Kingdom (β*_TheNetherlands_*=–.20, *P*<.001), women (β*_man_*=–.14, *P*<.001), and those imagining the unambiguous scenario (β*_unambiguous_*=.12, *P*<.001) reported the highest emotional intensity. Model 1 accounted for 8% of the variance (F_4,901_=18.28; *P*<.001). In model 2a (Table 3), it was observed that less emotionally stable (β=–.25, *P*<.001) and more agreeable (β=.12, *P*<.001) participants reported a higher emotional intensity. Model 2a additionally accounted for an additional 5% of the variance (F_9,901_=14.64; *P*<.001). Model 2b (Table 3) showed that those reporting more internalizing problems (β=.33, *P*<.001) and more resilient participants (β=.22, *P*<.001) reported a higher emotional intensity. Model 2b additionally accounted for 17% of the variance (F_7,901_=66.32; *P*<.001).

**Table 3:** Results of hierarchical regression analyses showing amount of variance in the emotional intensity dimension accounted for by country of residence, condition, gender, age, Big Five personality traits, DASS-21, aggression, and resilience.

Model		B^a^	SE	β^b^	*t*	*P* value	F^c^	*R*	*R* ^2^	Δ*R*^2d^
**1**										
	(Constant)	0.36	.11	—^e^	3.22	<.001	18.28	.28	.08	.08
	Country^f^	–0.40	.06	–.20	–6.14	<.001	—	—	—	—
	Condition^g^	0.23	.06	.12	3.60	<.001	—	—	—	—
	Gender^h^	–0.28	.06	–.14	–4.34	<.001	—	—	—	—
	Age	0	0	–.05	–1.39	.16	—	—	—	—
**2a**										
	(Constant)	0.21	.11	—	1.89	.06	14.64	.36	.13	.05
	Country^f^	–0.35	.06	–.18	–5.54	<.001	—	—	—	—
	Condition^g^	0.21	.06	.11	3.36	<.001	—	—	—	—
	Gender^h^	–0.17	.07	–.08	–2.52	.01	—	—	—	—
	Age	0	0	–.02	–.540	.59	—	—	—	—
	Extraversion	0.04	.05	.03	0.78	.44	—	—	—	—
	Agreeableness	0.19	.06	.12	3.05	<.001	—	—	—	—
	Conscientiousness	0.08	.06	.05	1.20	.23	—	—	—	—
	Emotional stability	–0.37	.06	–.25	–6.59	<.001	—	—	—	—
	Openness	–0.03	.07	–.01	–.390	.69	—	—	—	—
**2b**										
	(Constant)	–2.41	.24	—	–10.27	<.001	66.32	.49	.24	.17
	Country^f^	–0.29	.06	–.14	–4.85	<.001	—	—	—	—
	Condition^g^	0.18	.06	.09	3.01	.003	—	—	—	—
	Gender^h^	–0.32	.06	–.16	–5.42	<.001	—	—	—	—
	Age	0.01	0	.14	4.21	<.001	—	—	—	—
	Depression, anxiety, stress	0.44	.06	.33	7.26	<.001	—	—	—	—
	Aggression	0.09	.05	.08	1.80	.07	—	—	—	—
	Resilience	0.04	.01	.22	7.40	<.001	—	—	—	—

^a^B: unstandardized coefficient.

^b^β: beta standardized coefficient.

^c^F: F ratio.

^d^Δ*R*^2^: *R*^2^ change.

^e^Not applicable.

^f^Reference category: United Kingdom.

^g^Reference category: ambiguous situation.

^h^Reference category: women.

#### Proactive Versus Fight/Flight

More fight/flight reactions were reported by younger participants (β*_age_*=–.26, *P*<.001), by men (β*_man_*=.16, *P*<.001), and by participants responding to the unambiguous scenario (β*_unambiguous_*=.09, *P*=.006). Model 1 (Table 4) accounted for 9% of the variance (F_4,901_=22.59; *P*<.001). In model 2a (Table 4), it was observed that less agreeable (β=–.34, *P*<.001), less conscientious (β=–.19, *P*<.001), and less open (β=–.22, *P*<.001) but more extraverted (β=.07, *P*=.02) participants showed more fight/flight reactions. Model 2a accounted for an additional 32% of the variance (F_9,901_=68.57; *P*<.001). In model 2b (Table 4), it was observed that less resilient participants (β=–.19, *P*<.001) and participants with more internalizing problems (β*_DASS_*=.26, *P*<.001) and more aggression (β=.25, *P*<.001) reported more fight/flight reactions. Model 2b accounted for an additional 24% of the variance (F_7,901_=62.82; *P*<.001).

**Table 4:** Results of hierarchical regression analyses showing amount of variance in the proactive versus fight/flight reactions dimension accounted for by country of residence, condition, gender, age, Big Five personality traits, DASS-21, aggression, and resilience.

Model			B^a^		SE		β^b^	*t*		*P* value		F^c^		*R*		*R* ^2^		Δ*R*^2d^
**1**																		
	(Constant)	0.48		.11		—^e^		4.43	<.001		22.59		.30		.09		.09	
	Country^f^	0		.06		0		0	>.99		—		—		—		—	
	Condition^g^	0.17		.06		.09		2.73	.01		—		—		—		—	
	Gender^h^	0.31		.06		.16		4.85	<.001		—		—		—		—	
	Age	–0.02		0		–.26		–8.10	<.001		—		—		—		—	
**2a**																		
	(Constant)	0.08		.09		—		0.81	.42		68.57		.64		.41		.32	
	Country^f^	0.07		.05		.04		1.37	.17		—		—		—		—	
	Condition^g^	0.17		.05		.08		3.23	<.001		—		—		—		—	
	Gender^h^	0.07		.05		.03		1.21	.23		—		—		—		—	
	Age	–0.01		0		–.08		–2.92	<.001		—		—		—		—	
	Extraversion	0.10		.04		.07		2.44	.02		—		—		—		—	
	Agreeableness	–0.51		.05		–.34		–10.26	<.001		—		—		—		—	
	Conscientiousness	–0.31		.05		–.19		–6.08	0		—		—		—		—	
	Emotional stability	–0.05		.05		–.03		–0.99	.32		—		—		—		—	
	Openness	–0.41		.06		–.22		–7.29	<.001		—		—		—		—	
**2b**																		
	(Constant)	–0.33		.22		—		–1.51	.13		62.82		.57		.33		.24	
	Country^f^	0.12		.06		.06		2.17	.03		—		—		—		—	
	Condition^g^	0.14		.06		.07		2.26	.01		—		—		—		—	
	Gender^h^	0.20		.06		.10		3.58	<.001		—		—		—		—	
	Age	–0.01		0		–.07		–2.44	.02		—		—		—		—	
	Depression, anxiety, stress	0.34		.06		.26		6.04	<.001		—		—		—		—	
	Aggression	0.27		.05		.25		5.96	<.001		—		—		—		—	
	Resilience	–0.04		.01		–.19		–7.02	<.001		—		—		—		—	

^a^B: unstandardized coefficient.

^b^β: beta standardized coefficient.

^c^F: F ratio.

^d^Δ*R*^2^: *R*^2^ change.

^e^Not applicable.

^f^Reference category: United Kingdom.

^g^Reference category: ambiguous situation.

^h^Reference category: women.

#### Affective Versus Cognitive/Motivational

Model 1 (Table 5) showed that for older participants (β*_age_*=.21, *P*<.001), men (β*_man_*=.13, *P*<.001), and Dutch participants (β*_TheNetherlands_*=.08, *P*=.01), the cognitive/motivational reactions were more salient. The model accounted for 7% of the variance (F_4,901_=18.02; *P*<.001). Only emotional stability was a significant predictor of the salience of cognitive motivational reactions in model 2a (β=.14, *P*<.001). Model 2a (Table 5) accounted for an additional 2% of the variance (F_9,901_=10.57; *P*<.001). In model 2b, it was observed that more aggression (β=.13, *P*=.01) and less internalizing problems (β=–.32, *P*<.001) related to a comparatively higher salience of cognitive/motivational than affective reactions. Model 2b (Table 5) accounted for an additional 5% of the variance (F_7,901_=18.06; *P*<.001).

**Table 5:** Results of hierarchical regression analyses showing amount of variance in the affective versus cognitive/motivational dimension accounted for by country of residence, condition, gender, age, Big Five personality traits, DASS-21, aggression, and resilience.

Model		B^a^	SE	β^b^	*t*	*P*	F^c^	*R*	*R* ^2^	Δ*R*^2d^
**1**										
	(Constant)	–0.86	.11	—^e^	–7.81	<.001	18.02	.27	.07	.07
	Country^f^	0.16	.06	.08	2.52	.01	—	—	—	—
	Condition^g^	0.12	.06	.06	1.79	.07	—	—	—	—
	Gender^h^	0.25	.06	.13	3.95	<.001	—	—	—	—
	Age	0.02	0	.21	6.59	<.001	—	—	—	—
**2a**										
	(Constant)	–0.75	.12	—	–6.52	<.001	10.57	.31	.10	.02
	Country^f^	0.11	.06	.06	1.76	.08	—	—	—	—
	Condition^g^	0.13	.06	.07	2.06	.04	—	—	—	—
	Gender^h^	0.23	.07	.11	3.38	<.001	—	—	—	—
	Age	0.01	0	.18	5.33	<.001	—	—	—	—
	Extraversion	0.06	.05	.04	1.21	.23	—	—	—	—
	Agreeableness	–0.04	.06	–.03	–0.66	.51	—	—	—	—
	Conscientiousness	–0.04	.06	–.02	–0.58	.56	—	—	—	—
	Emotional stability	0.20	.06	.14	3.54	<.001	—	—	—	—
	Openness	0.04	.07	.02	0.51	.61	—	—	—	—
**2b**										
	(Constant)	–0.04	.25	—	–0.18	.86	18.06	.35	.12	.05
	Country^f^	0.13	.06	.07	2.03	.42	—	—	—	—
	Condition^g^	0.13	.06	.06	2.04	.41	—	—	—	—
	Gender^h^	0.26	.06	.13	4.08	<.001	—	—	—	—
	Age	0.01	0	.13	3.63	<.001	—	—	—	—
	Depression, anxiety, stress	–0.42	.07	–.32	–6.50	<.001	—	—	—	—
	Aggression	0.14	.05	.13	2.65	.01	—	—	—	—
	Resilience	0	.01	–.01	0.32	.75	—	—	—	—

^a^B: unstandardized coefficient.

^b^β: beta standardized coefficient.

^c^F: F ratio.

^d^Δ*R*^2^: *R*^2^ change.

^e^Not applicable.

^f^Reference category: United Kingdom.

^g^Reference category: ambiguous situation.

^h^Reference category: women.

## Discussion

### Internal Structure

The first and foremost goal of this study was to investigate the structure of emotional reactions in one of the most emblematic situations of cybersecurity breaches of the upcoming IoT devices—the hacking of one’s smart security camera—by looking at the full emotion process that can be elicited by this situation. Not a 1-dimensional but a 3-dimensional structure clearly emerges.

On the first dimension, all emotional reactions are loading positively. With the subjective experience items loading the highest on this dimension, this general intensity dimension can be best interpreted as a negative affectivity dimension, comparable to, for instance, the frequently used negative affectivity scale of the PANAS [13].

The second dimension represents the relative salience of proactive versus fight/flight action tendencies. This second dimension underlines the central status of action tendencies for the concept of emotion [44,45]. From an evolutionary perspective, emotion processes are phylogenetically shaped processes that quickly prepare the organism for action. However, depending on the concrete situation, these elicited action tendencies can be more or less constructive. In the new internet environment where we interact from a distance, acting aggressively or withdrawing are not adaptive reactions. One often does not know who is responsible and one’s life depends more and more on participating in this interconnected online world. Only the proactive tendencies to stop what is happening and to better protect oneself can be considered adaptive and lead to constructive results.

The third dimension describes the relative salience of cognitive/motivational versus affective (expression, bodily reactions, regulation, and feelings) features. Possibly, this finding can be linked to the different levels of consciousness with which appraisals can occur [46]. When the appraisals are made consciously, one can focus more on what one feels inclined to do and should do. When the appraisals are made unconsciously, the way the emotion is felt and expressed becomes more salient rather than what has elicited the emotion.

**Figure 1:**
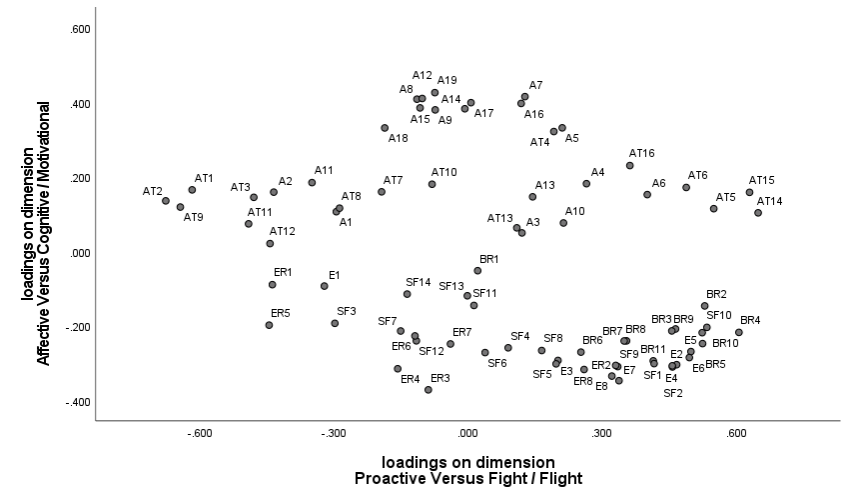
Plot of the loadings of the emotional reactions on the second and third dimension as a function of the emotion component to which they belong. A: appraisal; AT: action tendency; BR: bodily reaction; E: expression; SF: subjective feeling; ER: emotion regulation.

When the second and third dimensions are combined, a distinction emerges that has been referred to in the stress and coping literature as problem-focused versus emotion-focused coping [47] (Figure 1). The proactive tendencies in the upper-left quadrant correspond with problem-focused coping. The bodily reactions, subjective feelings, and expressions in the lower-left quadrant indicate that one is overwhelmed and regulation is required.

This 3-dimensional structure is highly replicable: exactly the same structure was found across the two versions of the security breach scenario, across the two countries with their respective languages, and across the two genders (Multimedia Appendix 1).

### Predictors of Emotion Dimensions

The second goal was to explore whether personality characteristics predict the empirically identified emotion dimensions, and, if that is the case, which ones (Tables 3-5). The general finding is that the broad personality characteristics from both personality models relate differentially to the 3 emotion dimensions, which confirms that these emotion dimensions are indeed each capturing valid aspects of the emotion processes.

#### Big Five Personality Model

The two most predictive personality traits are emotional stability and agreeableness. In line with the well-documented negative relationship between emotional stability and negative affectivity [22-24], we observed that emotionally stable participants scored lower on the general emotion dimension and reported a higher salience of the affective components. Agreeable participants showed more proactive action tendencies and tended also to score a bit higher on the general emotion dimension. It is possible that agreeable people, who value warm interpersonal relationships, appraise negatively intended actions by others, like hacking, as more relevant while at the same time are less inclined to react aggressively, which frees more energy to deal constructively with the situation. Moreover, agreeable people are more likely to use cognitive restructuring and problem-solving approaches [22-24]. Conscientiousness and openness only predicted proactive tendencies. Being diligent, efficient, and orderly, which are characteristics of conscientious people, might help to focus on the action tendencies that can provide support in effectively dealing with the situation. The relationship with the personality trait openness was a bit less self-evident. As IoT is a recent and fast-developing field, people who are more curious and open are possibly more likely to understand the full implications of cybersecurity breaches and act accordingly. Extraversion, which has been found in the literature to be predictive of positive but not negative affectivity [22-24], was virtually unrelated to the emotion dimensions (with the exception of a very small although statistically significant relationship with fight/flight tendencies, probably due to the fact that extravert people tend to express their emotions more) [48].

#### Resilient/Overcontrolled/Undercontrolled Personality Type Model

Internalization problems, which are important characteristics of an overcontrolled personality type, predicted a higher general emotional intensity, more fight/flight tendencies, and a comparatively higher salience of the affective components. This finding indicates that people who are already vulnerable do not succeed in adequately dealing with the emotional experience. Interestingly, resilience, a characteristic of well-functioning people [43], not only predicts more proactive tendencies but also a higher general intensity of emotional reactions and a higher salience of the affective components. Possibly because resilient people can cope better with stressors, they are less defensive and more willing to appraise the seriousness of the situation and accept their own emotional reactions. Finally, aggression, as an indicator of an undercontrolled personality type, is especially predictive of fight/flight action tendencies and relates to a slightly higher salience of the cognitive/motivational components. People who are high on aggression are more willing to blame others and are primed on aggressive reactions [49,50].

#### Gender and Age

In addition to the personality predictors, we also found that gender and age played a role. Women had a tendency to have more emotionally intense and affective reactions, while men were more likely to show fight/flight reactions. This is in line with earlier findings that females generally have more intense emotional reactions [25-28], experience more emotions in situations of cyberbullying [51], and have more anxiety in situations of hacking [52] and that males tend to react more aggressively [53].

We also found that older individuals were more prone to have proactive and cognitive/motivational reactions, which fits the observation that older individuals have less negative affective experiences and healthier emotion regulation strategies [29-32]. Additionally, intra-individual differences in emotional reactions to cybersecurity breaches are organized and structured in exactly the same way for males and females (Multimedia Appendix 1).

#### Ambiguous/Nonambiguous Conditions

While the two cybersecurity scenarios showed exactly the same 3-dimensional structure of emotional reactions, quantitative differences were observed, with the nonambiguous situation eliciting more intense and more fight/flight emotional reactions. The nonambiguous situation is possibly experienced more as though one is confronted with a natural person in real life. The situation becomes more relevant for one’s goals and elicits more fight/flight action tendencies rather than the more adaptive proactive reactions.

#### Country

Finally, while the emotion structure is the same in the two countries, we observed less intense reactions and a higher salience of cognitive/motivational reactions in the Dutch as compared with the UK sample. A speculative explanation could be that the emblematic example of the hacking of a smart security camera has received more media coverage in the United Kingdom [54-57] than in the Netherlands, which has made these scenarios more emotionally salient in the United Kingdom.

### Principal Findings

In this study, only the direct emotional reactions to a cybersecurity breach scenario have been studied. A question for future research is whether and to what extent these immediate emotional reactions set the stage for further mental health problems. Being exposed to hacking has been linked to psychopathology [58-61] in the literature and even to suicide in the media [9]. Based on the discovered emotion structure, very different dynamics can be predicted with respect to the role that a hacking situation will play in a person’s life in the longer run.

Those with intense emotional reactions, fight/flight action tendencies, and salient affective components are probably more likely to stay confronted with the situation and its negative ramifications. They experience the situation as emotionally highly relevant, but they tend to react in a way that does not resolve the challenges created by the problem. Moreover, they are additionally confronted with affective reactions that need to be regulated and thus require extra energy. This combination can be considered the psychologically least adaptive reaction, which sets the stage for further mental health complaints.

Those who have no or little negative emotional reactions can only be partially considered better off. They do not have to deal with the negative emotional reactions themselves but also lack the inherent pressure created in the emotion process to take action. Emotions are relevance detectors [5]. Appraising the situation as threatening with its ensuing negative emotional reactions can motivate appropriate action and can therefore be considered adaptive. This interpretation is also supported by the finding that resilient people score higher on the general intensity dimension.

The most adaptive emotional reaction can be considered to be a negative emotional reaction in which the proactive and constructive action tendencies and cognitive-motivational components are the most salient. Such a reaction pattern implies that the seriousness of the situation is adequately appraised and thus that the emotions play their role as relevance detectors. At the same time, actions are prepared that maximize an effective resolution of the situation without the person being overwhelmed by the affective reactions.

### Limitations

One of the limitations of this study is that the causal conclusions about the long-term mental health consequences of a cybersecurity breach cannot be investigated with a scenario methodology based on anticipated emotional experiences. However, as experimental research of real emotional experiences is impossible or at least highly limited in this area due to ethical considerations (it is unethical to actually invade the privacy of people by hacking their security camera), scenarios offer an ethically viable and direct way to study the structure of emotional reactions in this uncharted domain. As this study was conducted in Western Europe, further cultural generalizability is yet to be demonstrated. Future research can also study the ecological validity, generalizability, and long-term mental health implications of these findings. Another limitation is the use of self-assessment instruments. While some emotion components can only be studied through self-assessment (like subjective feelings and cognitive appraisals), other components can be studied by objective data (like psychophysiological and expressive changes). In future research, it would be interesting to complement self-reported data with such objective data.

### Conclusion

With the increasing interconnections through the internet and especially the recent development of IoT, people are much more at risk of experiencing cybersecurity breaches. Becoming a victim of cybersecurity breaches, with possibly far-reaching consequences for one’s personal and professional life, is becoming more and more likely. When all components of the emotion processes elicited by such cybersecurity breaches are investigated, a replicable 3-dimensional structure emerges that goes beyond the well-known negative affectivity dimension. These dimensions relate differentially to broad personality characteristics, which further validates the need for a multidimensional representation. Depending on the position of the emotional reaction on these three dimensions, very different predictions can be made about the long-term mental health implications of hacking experiences. With this study, a key process that links the occurrence of a cybersecurity breach situation with possible long-term mental health effects has been mapped out.

## Appendix

Multimedia Appendix 1 Supplement.

Multimedia Appendix 2 Cybersecurity GRID.

## Abbreviations

DASS-21: Depression, Anxiety and Stress Scale–21 Item
IoT: Internet of Things
IPIP-50: International Personality Item Pool 50
PANAS: positive and negative affect schedule
