# Supplementary material for: Emotional Reactions to Cybersecurity Breach Situations: Scenario-Based Survey Study
Source: J Med Internet Res. 2021 May 12;23(5):e24879. doi: 10.2196/24879 (PMC8156130; doi:10.2196/24879)
Supplement: Multimedia Appendix 1 [file jmir_v23i5e24879_app1.pdf]

Multimedia Appendix 1. Supplemental materials.

Table S1

Results from Principal Component Analyses of the GRID questionnaire

GRID items	Component loading		
	1	2	3
AT14 I would want to destroy whatever was close.	.32	**.65**	.10
AT15 I would want to take revenge.	.36	**.63**	.16
BR4 I would have pain in the chest.	.49	**.61**	-.22
AT5 I would want to isolate myself physically.	.44	**.55**	.11
BR2 I would be dizzy.	.52	**.53**	-.15
SF10 I would feel ashamed.	.48	**.53**	-.20
BR10 I would have a dry mouth.	.54	**.52**	-.22
BR5 I would sweat (whole body).	.56	**.52**	-.25
E5 I would slouch (shoulders down, head down, hands down).	.48	**.50**	-.27
E6 I would cover my face with my hands.	.48	**.50**	-.29
AT6 I would want to isolate myself in the virtual world (e.g., close my online accounts)	.45	**.49**	.17
E2 I would have tears in my eyes.	.55	**.47**	**-.30**
BR3 I would be shaking.	.61	**.46**	-.21
BR9 I would have goosebumps.	.53	**.46**	-.21

E4 I would have a trembling voice.	.61	**.46**	**-.31**
SF2 I would experience the emotional state for a long time.	.61	**.46**	**-.31**
SF1 I would be in an intense emotional state.	.63	**.42**	**-.30**
BR11 My body would become hot (puff of heat, cheeks or chest).	.58	**.41**	-.29
A6 I would think “I cannot do much about that situation."	.28	**.40**	.15
AT16 I would want to find and punish the attacker.	.46	**.36**	.23
BR7 My breathing would be faster.	.64	**.35**	-.24
BR8 My muscles would be tense.	.63	**.35**	-.24
SF9 I would feel sad.	.61	**.33**	**-.31**
ER2 I would want people to comfort me.	.56	**.33**	**-.31**
SF3 I would feel anxious.	.65	**-.30**	-.19
E1 I would frown.	.49	**-.32**	-.09
A11 I would think “There is a problem with the device or the account."	.34	**-.35**	.18
A2 I would think “I wonder whether something is wrong with the device/account."	.34	**-.43**	.16
AT12 I would want to protect my device.	.45	**-.44**	.02
ER1 I would want to ask others to help me in solving the problem.	.51	**-.44**	-.09
ER5 I would try to make the best out of the situation.	.29	**-.45**	-.20
AT3 I would want to protect myself.	.52	**-.48**	.14
AT11 I would want to reset my device.	.42	**-.49**	.07
AT1 I would want to stop what was happening.	.43	**-.62**	.16
AT9 I would want to find a solution and fix the problem.	.34	**-.64**	.12

AT2 I would want to regain control over the device/account.	.44	**-.68**	.14
A19 I would think “It is not safe that this device is connected to the Internet."	.58	-.07	**.43**
A7 I would think “My trust is betrayed."	.58	.13	**.42**
A12 I would think “It is happening because someone is trying to hack and take control over my count	.56	-.10	**.41**
A8 I would think “My security could be jeopardized."	.60	-.11	**.41**
A16 I would think “Someone could destroy my data."	.59	.12	**.40**
A17 I would think “Someone could use my data to harm me."	.63	.01	**.40**
A14 I would think “I could lose personal information, data and documents."	.58	-.01	**.38**
A9 I would think “The security of people close to me could be jeopardized."	.61	-.07	**.38**
A15 I would think “Someone may have access to my private information."	.65	-.11	**.38**
A5 I would think “I cannot use the device or service anymore."	.45	.21	**.33**
A18 I would think “Similar situations might happen again in the future."	.53	-.19	**.33**
AT4 I would want to stop using devices that are connected to the Internet.	.48	.19	**.32**
SF5 I would feel panic.	.73	.20	**-.30**
ER8 I would have trouble concentrating.	.62	.26	**-.32**
ER4 I would try to suppress my feelings and control myself.	.52	-.16	**-.32**
E8 I would be walking around nervously.	.60	.32	**-.34**
E7 I would be restless (touching face, hair, biting nails, nervously kicking with legs).	.58	.34	**-.35**
ER3 I would try to calm myself down (e.g., by breathing deeply)	.59	-.09	**-.37**
A4 I would think “My data are not available anymore"	.41	.27	.18
AT10 I would want to report the situation (e.g., to the police or to the Internet provider).	.54	-.08	.18

AT7 I would want to change my privacy settings.	.55	-.19	.16
A13 I would think “It is happening because a household member did something with the device".	.36	.14	.15
AT8 I would want to save my data.	.48	-.29	.12
A1 I would think “I do not know what is happening.”	.37	-.29	.11
A10 I would think “It is happening because I did something wrong."	.37	.21	.08
AT13 I would want to swear and curse.	.47	.11	.06
A3 I would think “I am confused."	.39	.12	.05
BR1 I would have stomach discomfort.	.59	.02	-.05
SF13 I would feel surprised.	.54	.00	-.12
SF14 I would feel uncomfortable.	.67	-.14	-.12
SF11 I would feel angry.	.67	.01	-.15
SF7 I would feel worried.	.70	-.15	-.21
ER6 I would try to see the positive side of the situation	.16	-.12	-.23
SF12 I would feel frustrated.	.63	-.12	-.24
ER7 I would not stop thinking and analyzing the situation.	.57	-.04	-.25
SF4 I would feel afraid.	.72	.09	-.26
BR6 My heartbeat would be faster.	.66	.25	-.27
SF8 I would feel powerless.	.63	.16	-.27
SF6 I would feel upset.	.70	.04	-.27
E3 I would speak louder.	.57	.20	-.29

Table S2

Total Variance Explained

Component	Initial Eigenvalues			Extraction Sums of Squared Loadings		
	Total	% of Variance	Cumulative %	Total	% of Variance	Cumulative %
1	23.372	30.753	30.753	23.372	30.753	30.753
2	8.959	11.789	42.541	8.959	11.789	42.541
3	3.866	5.086	47.628	3.866	5.086	47.628
4	2.608	3.432	51.060			
5	2.014	2.650	53.710			
6	1.797	2.365	56.075			
7	1.491	1.962	58.037			
8	1.406	1.850	59.887			
9	1.269	1.669	61.556			
10	1.169	1.538	63.094			

Extraction Method: Principal Component Analysis.

Table S3

Cronbach alpha for scales calculated separately for UK and NL sample

	**UK**	**NL**
	α	α
Extraversion	.84	.85
Agreeableness	.83	.83
Conscientiousness	.79	.78
Emotional Stability	.84	.81
Openness	.70	.73
DASS	.97	.96
Agression	.92	.92
Resilience	.79	.77

Table S4

Table S4. Congruence measures (Tucker's phi coefficients) comparing the principal component structure in different conditions with the overall principal component structure.

Condition	Emotion dimension		
	1	2	3
UK	.999	.981	.985
NL	.999	.986	.979
Male	.998	.993	.983
Female	.998	.994	.982
Ambiguous	.999	.994	.993
Non-Ambiguous	.998	.986	.991

Supplement S5

Information presented to all participants.

Internet of Things refers to all every day physical objects and/or devices that are connected to the internet and/or other devices. Examples are smart cameras, smart speakers, smart vacuum cleaners, smart light bulbs, smart TV's, smart watches etc.

In this study we want to focus on one frequently used IoT device, which is the smart security camera.

A smart security camera allows you to have access to your security camera even when you are not at home. You can control the smart camera through your smartphone or through a control device specific to your camera. At any moment, you can have access to the live video stream as well as to recorded history. The camera has an opaque mechanical shutter, in front of the lens, which you can open or close, reassured that no video stream is being recorded when the shutter is closed. We would like to ask you to imagine yourself in the following situation and imagine how you would feel if you experience it.

Scenario 1

Imagine that you bought a smart security camera for your home. After some time, you notice that the shutter on your smart security camera starts opening and closing without your instruction, several times for a few minutes, then it stops for a minute and starts again opening and closing several times and then it stops.

Scenario 2

Imagine that you bought a smart security camera for your home. After some time, you notice that the shutter opens without your instruction and the camera rotates towards you and then starts following your movement.
