# Supplementary material for: Emotional Reactions to Cybersecurity Breach Situations: Scenario-Based Survey Study
Source: J Med Internet Res. 2021 May 12;23(5):e24879. doi: 10.2196/24879 (PMC8156130; doi:10.2196/24879)
Supplement: Multimedia Appendix 2 [file jmir_v23i5e24879_app2.pdf]

Cybersecurity GRID

We would like you to imagine as vividly as possible the situation that is described, as well as how you would feel at that moment. Please indicate on the items below the extent they would represent your emotional experience at that moment, using the following 7-point response scale:

1 Strongly disagree
2 Disagree
3 Somewhat disagree
4 Neither agree nor disagree
5 Somewhat agree
6 Agree
7 Strongly agree

APPRAISAL

These items represent what you would think, want to do, feel in your body, express, subjectively feel, and how you would regulate yourself.

A1 I would think “I do not know what is happening”.
A2 I would think “I wonder whether something is wrong with the device".
A3 I would think “I am confused".
A4 I would think “My data are not available anymore".
A5 I would think “I cannot use the device or service anymore".
A6 I would think “I cannot do much about that situation".
A7 I would think “My trust is betrayed".
A8 I would think “My security could be jeopardized".
A9 I would think “The security of people close to me could be jeopardized".
A10 I would think “It is happening because I did something wrong".
A11 I would think “There is a problem with the device".
A12 I would think “It is happening because someone is trying to hack and take control over my device".
A13 I would think “It is happening because a household member did something with the device".
A14 I would think “I could lose personal information, data and documents".
A15 I would think “Someone may have access to my private information".
A16 I would think “Someone could destroy my data".
A17 I would think “Someone could use my data to harm me".
A18 I would think “Similar situations might happen again in the future".
A19 I would think “It is not safe that this device is connected to the Internet".

ACTION TENDENCIES

These items represent what you would think, want to do, feel in your body, express, subjectively feel, and how you would regulate yourself.

AT1 I would want to stop what is happening.
AT2 I would want to regain control over the devices.
AT3 I would want to protect myself.
AT4 I would want to stop using devices that are connected to the Internet.
AT5 I would want to isolate myself physically.
AT6 I would want to isolate myself in the virtual world (e.g., close my online accounts).
AT7 I would want to change my privacy settings.
AT8 I would want to save my data.
AT9 I would want to find a solution and fix the problem.
AT10 I would want to report the situation (e.g., to the police or to the internet provider).
AT11 I would want to reset my device.
AT12 I would want to protect my device.
AT13 I would want to swear and curse.
AT14 I would want to destroy whatever was close.
AT15 I would want to take revenge.
AT16 I would want to find and punish the attacker.

BODILY RESPONSE

These items represent what you would think, want to do, feel in your body, express, subjectively feel, and how you would regulate yourself.

BR1 I would have stomach discomfort.
BR2 I would be dizzy.
BR3 I would be shaking.
BR4 I would have pain in the chest.
BR5 I would sweat (whole body).
BR6 My heartbeat would be faster.
BR7 My breathing would be faster.
BR8 My muscles would be tense.
BR9 I would have goose bumps.
BR10 I would have dry mouth.
BR11 My body would become hot (puff of heat, cheeks or chest).

EXPRESSION

These items represent what you would think, want to do, feel in your body, express, subjectively feel, and how you would regulate yourself?

E1 I would frown.
E2 I would have tears in my eyes.
E3 I would speak louder.
E4 I would have a trembling voice.
E5 I would slouch (shoulders down, head down, hands down).
E6 I would cover my face with my hands.
E7 I would be restless (touching face, hair, biting nails, nervously kicking with legs).
E8 I would be walking around nervously.

SUBJECTIVE FEELING

These items represent what you would think, want to do, feel in your body, express, subjectively feel, and how you would regulate yourself.

SF1 I would be in an intense emotional state.
SF2 I would experience the emotional state for a long time.
SF3 I would feel anxious.
SF4 I would feel afraid.
SF5 I would feel panic.
SF6 I would feel upset.
SF7 I would feel worried.
SF8 I would feel powerless.
SF9 I would feel sad.
SF10 I would feel ashamed.
SF11 I would feel angry.
SF12 I would feel frustrated.
SF13 I would feel surprised.
SF14 I would feel uncomfortable.

EMOTION REGULATION

These items represent what you would think, want to do, feel in your body, express, subjectively feel, and how you would regulate yourself.

ER1 I would want to ask others to help me in solving the problem.
ER2 I would want people to comfort me.
ER3 I would try to calm myself down (e.g., by breathing deeply).
ER4 I would try to suppress my feelings and control myself.
ER5 I would try to make the best out of the situation.
ER6 I would try to see the positive side of the situation.
ER7 I would not stop thinking and analyzing the situation.
ER8 I would have trouble concentrating.
